# Cost-effectiveness of exercise referral schemes: a systematic review of health economic studies

**DOI:** 10.1093/eurpub/ckab189

**Published:** 2021-12-04

**Authors:** Amber Werbrouck, Masja Schmidt, Koen Putman, Jan Seghers, Steven Simoens, Nick Verhaeghe, Lieven Annemans

**Affiliations:** 1 Interuniversity Centre for Health Economics Research (I-CHER), Department of Public Health and Primary Care, Ghent University, Corneel Heymanslaan 10, 9000 Ghent, Belgium; 2 Department of Pharmaceutical and Pharmacological Sciences, KU Leuven, Herestraat 49, O&N2 Bus 521, Leuven 3000, Belgium; 3 Interuniversity Centre for Health Economics Research (I-CHER), Department of Public Health, Vrije Universiteit Brussel, Laarbeeklaan 103, 1090 Brussels, Belgium; 4 Department of Movement Sciences, KU Leuven, Tervuursevest 101 box 1500, Leuven 3001, Belgium; 5 HIVA Research Institute for Work and Society, KU Leuven, Parkstraat 47 Box 5300, Leuven 3000, Belgium

## Abstract

**Background:**

This systematic review aimed to provide an overview of the existing literature on cost-effectiveness of exercise referral schemes (ERSs).

**Methods:**

A systematic search was performed in MEDLINE, EMBASE, EconLit, Web of Science and PsycINFO. Main inclusion criteria were: (1) insufficiently active people; (2) ERSs and (3) full health economic evaluations. No publication year limits were applied. The methodological quality was assessed independently by two reviewers using the Consensus Health Economic Criteria (CHEC) checklist.

**Results:**

Fifteen eligible publications were retrieved, presenting results of 12 different studies. Compared with usual care, ERSs were found to be cost-effective in a majority of the analyses, but with modest health gains and costs per individual. These cost-effectiveness results were also sensitive to small changes in input parameters. Two studies found that ERSs combined with a pedometer/accelerometer are cost-effective, compared with usual ERS practice. Two other studies found that an ERS with phone support and an ERS with face-to-face support might be equally effective, with similar costs.

**Conclusion:**

Although the literature demonstrated that ERSs could be cost-effective compared with usual care, these results were not robust. Based on a small number of studies, ERSs could be optimized by using tracking devices, or by providing a choice to the participants about the delivery mode. There is need for clarity on the effectiveness of and attendance to ERS, as more certainty about these key input parameters will strengthen health-economic evidence, and thus will allow to provide a clearer message to health policy-makers.

## Introduction

In Europe, almost one-third of the adult population is insufficiently active.^1^ It is well established that physical inactivity is associated with the development and progression of several chronic conditions, including diabetes and cardiovascular diseases.^2^^,^^3^ In addition, physical activity (PA) and exercise have the potential to prevent and/or manage several other conditions, including chronic pain,^4^ chronic renal failure^5^ and depression.^6^ Hence, physical inactivity is one of the top modifiable risk factors for several lifestyle-related conditions, with high potential health gains as well as cost savings.^7^^,^^8^

Health care policy makers have been encouraging the population to develop and maintain an active lifestyle via different approaches. For example, several recommendations and guidelines have been developed.^9^^,^^10^ In this regard, several countries including the UK, Australia, Sweden, the USA and Belgium have also introduced exercises referral schemes (ERSs), a programme in which general practitioners or other primary care professionals can refer to a third party service in order to physically activate people who are sedentary or insufficiently active.^11–13^ Despite the popularity of ERSs across industrialized countries, some concerns have been raised. First, the evidence on its effectiveness is still inconsistent.^12^ Second, ERSs specifically aims to decrease health inequity by targeting socioeconomic disadvantaged people, but whether or not this goal is actually achieved by these programmes, is also subject to further research.^14^

Next to effectiveness and equity, several frameworks indicate that multiple aspects should be considered for implementation and evaluation of policy measures such as ERS. For example, the Institute of Medicine states that, among other things, health care systems should also strive for efficiency.^15^ The latter can be assessed by health economic evaluations.^16^ Several health economic evaluations of ERSs are available, but systematic reviews can be of added value as they summarize and synthesize existing evidence. We have identified a systematic review that included ERSs and other PA interventions in primary care.^17^ Additionally, three systematic reviews were retrieved that aimed to assess effectiveness as well as cost-effectiveness of ERSs.^18–20^ In these four reviews, only a small number of original studies on cost-effectiveness of ERSs were included, which can be explained by different primary aims of these reviews. Consequently, synthesis of the results related to cost-effectiveness of ERSs was often very brief,^17^^,^^18^^,^^20^ or limited to studies that considered a population with an underlying health condition.^19^ As most ERSs aim to target insufficiently active people, with or without any medical condition, there is a need to review all relevant and recent evidence. Therefore, the aim of this systematic review was to assess existing literature on cost-effectiveness of ERSs.

## Methods

The ‘Preferred Reporting Items for Systematic Reviews and Meta-Analyses’ (PRISMA) statement was used to structure this systematic review.^21^ Other guidelines, specifically focused on the preparation of a systematic review of health economic evaluations, have been consulted.^22–24^

For this systematic review, an ERS was defined as comprising three core components: (1) referral by a primary care healthcare professional to a third party service provider, designed to increase PA or exercise; (2) PA or exercise programme tailored to individual needs and (3) initial assessment and monitoring throughout the programme. The ERS and the PA or exercise programme had to be more intensive than simple advice and needed to include at least one form of counselling (in person or by telephone, by use of written materials and/or by supervised exercise training).^12^

A literature search strategy was developed for MEDLINE (via PubMed), and adapted for EMBASE (via embase.com), Web of Science Core Collection (via Web Of Science), EconLit (via ProQuest) and PsycINFO (via ProQuest). The reference lists of included studies were hand searched for potential relevant articles. Systematic reviews and protocols of health economic evaluations were collected separately, as a source for additional references.

Search strings were developed based on exploration of databases and previous reviews. The key concepts translated into search strings were: (1) ERSs and (2) full health economic studies. The first search string was based on existing reviews and further developed in consultation with a clinical expert (J.S.). The search string takes into account the large variety in terminology for ERSs. The second search string was based on search filters of the National Health Service Economic Evaluation Database,^25^ with specific attention to the identification of full health-economic evaluations. Search strategies of all electronic databases are presented in the Supplementary Material.

Eligibility criteria were defined *a priori* for study selection, see table 1. The PICO (i.e. Population, Intervention, Comparator and Outcome) strategy was applied to describe the criteria. As sedentary behaviour or insufficient PA has been defined and/or measured differently,^3^ and the aim was to include all studies that assessed people who are insufficiently active or sedentary, no definition for these constructs were predefined for inclusion in this systematic review.

**Table 1:** Eligibility criteria

	Inclusion criteria	Exclusion criteria
Population	Sedentary or insufficiently active people, of all ages, with or without any diagnosed condition	Active people (e.g. training schemes for athletes)
Intervention	Any type of ERSs	Programmes that are limited to simple advice, exercise programmes that are not tailored/individualized, or exercise programmes in the context of rehabilitation or (recovery from) injury.
Comparator	All comparators (no intervention, standard care or any other intervention)	/
Outcomes	/	/
Study design	Full health-economic evaluations	Partial health-economic evaluations
		Systematic reviews, reports, commentaries, congress abstracts, protocols and animal studies
Context	All settings	
Language	English, French, German or Dutch	

*Note*: “/” indicates that no criterion was defined for this aspect.

Two reviewers (A.W. and M.S.) independently screened the titles and abstracts yielded by the search, blinded to each other’s decision, using the web application Rayyan.^26^ Selection was based on the eligibility criteria (see table 1). Full texts were obtained for all eligible records. Second, screening on full text was executed by two reviewers (A.W. and M.S.), against the same eligibility criteria. During the second screening round, reasons for exclusion were noted. Additional information was searched to resolve ambiguities about eligibility. Disagreement about inclusion or exclusion was resolved by discussion; otherwise, a third reviewer was consulted. Multiple publications of the same study were linked.

One reviewer (A.W.) extracted the data. A data extraction sheet was developed in Microsoft Excel based on an existing template^24^ and adjusted for the objectives of this review. The following information was extracted from the original article or additional information sources: study identification, funding, general study characteristics, methodological approach (i.e. model-based or within-trial) and characteristics, results and author’s conclusions. The principal outcome measures were health economic outcomes included (incremental) health outcomes, (incremental) cost outcomes and (incremental) cost-effectiveness ratios (ICERs).

Two reviewers (A.W. and M.S.) independently evaluated the quality of included studies to assess risk of bias using the Consensus on Health Economic Criteria (CHEC) checklist.^27^ See the Supplementary Material for the full assessment instructions, as well as interpretations and adaptations of the checklist.

To facilitate comparison across studies, the following adjustments and/or interpretations were made: (1) all incremental costs and health outcomes are presented per 1,000 participants; (2) different currencies were converted to euros (reference year: 2019; reference country: Belgium)^28^; (3) control groups were categorized into usual care (‘usual care’, ‘care as usual’, ‘current practice’, ‘no additional care’, etc.) and enhanced usual care [e.g. simple (one-time) advice, or written information]; (4) types of studies were categorized into within-trial evaluations and model-based evaluations^16^ and (5) perspectives were categorized into^29^ third party payer perspective, total health care payer perspective and societal perspective.

## Results

**Figure 1:**
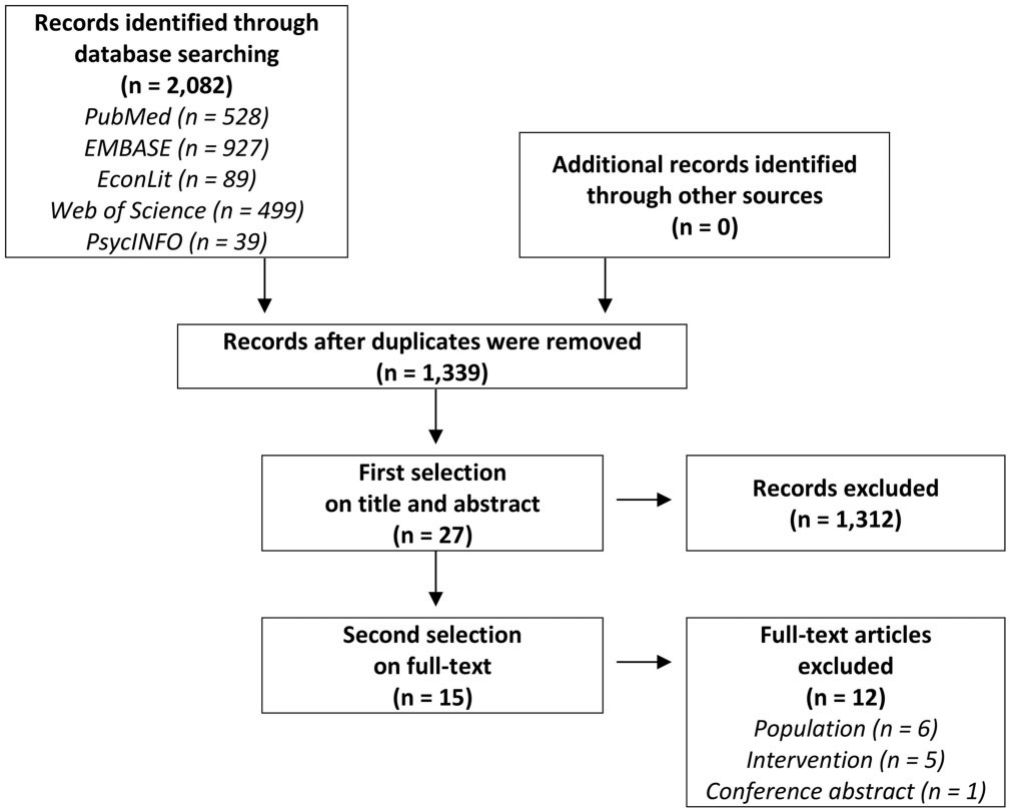
Flow chart of study selection. n, number

Starting from 2,082 records, a total of 15 publications (references a–o, see Supplementary Material) presenting results of 12 different studies were included (figure 1). Three publications reported the same results of the ERS across several UK-countries (k–m) and 2 publications reported the same results of the National ERS in Wales (a, b). These publications were linked and considered as one study.

Three studies reported different comparisons and/or different outcomes that could be included in this systematic review (c, e, o), resulting in a total of 17 analyses. An overview of the analyses is provided in table 2.

**Table 2:** Evidence table

	Study characteristics						Included alternatives			Incremental analyses			
	Country^A^	Mean age^B^; sample size	Med. cond.?	Time hor.	Perspec tive	Disc. rates	Intervention	Comparator	Other inter ventions	Incremental cost per 1,000 participants^C^: currency, ref.Y [€, 2019]	Incremental effect per 1,000 participants^C^ (outcome measure)	ICER^C^	CE?^D^
**Within-trial studies**													
Murphy, 2012 (a); Edwards, 2013 (b)	GBR	52Y *n* = 798	No	1Y	[i]		NERS	UC+	No	£, 2008: 327,000 [461,548]	QALY (EQ-5D-3L): 27	£[€]/QALY: 12,111 [17,094]	Yes
Leung, 2012 (c)	NZL	74Y *n* = 270	No	^*^1Y	[i]/[ii]		GRx + pedometer	GRx	No	NZL$, 2008: [i]: 57,000 [38,429] [ii]: 118,000 [79,555] [ii]+: −79,000 [53,262]	QALY (EQ-5D-3L): ^*^35 = n.s.	£[€]/QALY: [i]: 3,105 [2,093] [ii]: 3,500 [2,360] [ii]+: dominant	Yes
											30 min weekly leisure walking (Auckland Heart Study PA quest.): n.m.	£[€]/30 min weekly leisure walking [i]: 115 [78] [ii]: 130 [88] [ii]+: dominant	Yes
Hawkins, 2019 (d)	GBR	56.6Y *n* = 156	No	1Y	[i]		NERS + accelerometry	NERS	No	£, 2017: 386,000 [469,975]	QALY (EQ-5D-5L): 70 = n.s.	£[€]/QALY: ^*^5,514 [6,714]	Yes
Ewald, 2018 (e)	AUS	57Y *n* = 203	No	1Y	[i]		PA coaching (5× FTF, or 1× FTF + 4× phone)	UC+	No	AUD$, ^*^ 2015: 245,000 [151,580]	QALY (AQOL): n.s.		No
											Steps per day (pedometer): 1,002,000	AUD$[€]/1000 steps per day: 245 [151]	Yes
							PA coaching (1× FTF + 4× phone)	PA coaching (5× FTF)	No	AUD$, ^*^ 2015: n.m.	QALY (AQOL): ^*^n.s.		No
											Steps per day (pedometer): 619 = n.s.		No
Elley, 2004 (f)	NZL	57.2Y (I1) *n* = 878	No	1/4Y	[i]		GRx	UC	No	NZL$, 2001: 170,430 [137,868]	% persons active (n.m.): ^*^9.7	NZL$[€]/person made active: 1,756	Yes
Romé, 2009 (g)	SWE	55.2Y (I1) *n* = 525	Yes	1/3Y	[iii]		PA in prescription	UC+	No	SEK, 2007: ITT: ^*^3,437,000 [395,019] PP: ^*^5,899,000 [677,980]	MET min/week (IPAQ): n.s. % inactivity (TTM): n.s. Meters (6MWT): n.s.		No
Foley, 2011 (h)	NZL	59Y (I1) *n* = 3275	No	1Y	[i]		GRx (FTF, group)	GRx (phone)	No	NZL$, 2007: 6,070 [4,251]	Days/week active: 990 Health and well-being: n.s. Client satisfaction: n.s.		No
Elley, 2011 (i)	NZL	59.1Y (I1) *n* = 1089	No	1Y/2Y	[iii]		Enhanced GRx (1× FTF + 5× phone; women only)	UC	No	NZL$, 2008: 1Y: 86,940 [58,615] 2Y: 94,290 [63,570]	% persons active (NZL PA quest.): 1Y: 14 2Y: 7	NZL$[€]/person made active: 1Y: 638 [430] 2Y: 1,416 [2]	Yes
**Model-based studies**													
Dalziel, 2005 (j)	NZL	58Y	No	LT	[i]	5%	GRx	UC	No	NZL$, 2001: 161,000 [130,240]	QALY (SF-36): ^*^79	NZL$[€]/QALY: 2,053 [1,661]	Yes
Anokye, 2011 (k); Pavey, 2011 (l); Trueman, 2012 (m)	GBR	40–60Y	No	LT	[i] [ii]	3.5%	ERS (leisure centre- based)	UC	No	£, 2010: 169,540 [231,931]	QALY (EQ-5D-3L): 8 Physically active: 3900 people Healthy: 152 people Reduced anxiety: ES 0.219 Avoided T2DM cases: 86 Reduced absenteeism: ES 0.19 …	£[€]/QALY: 20,876 [28,559]	Yes
Campbell, 2015 (n)	GBR	50Y	No	LT	[i]	1.5%	ERS	UC	No	£, 2012: 225,400 [297,887]	QALY (^*^EQ-5D): 3	£[€]/QALY: 76,059 [100,520]	No
Cobiac, 2009 (o)	AUS	60Y	No	LT	[ii]	3%	GP referral (FTF)	UC	Yes	^E^ AUS$, 2003: 140,000,000 [121,420,000]	^E^ DALYs averted: 1,900	AUS$[€]/DALY: 79,000 [68,515]	No
							GP prescription (phone)	UC	Yes	AUS$, 2003: 81,000,000 [70,250,000]	DALYs averted: 7,100	AUS$[€]/DALY: 11,000 [9,540]	Yes

*Notes*: The studies are categorized as within-trial economic evaluations and model-based economic evaluations. The order within each category was based on the outcome measure that was used in the study (e.g. QALYs). (a–o), see Supplementary Material for the references of the included studies. [€, 2019], original costs converted to euros, reference year 2019, reference country Belgium; [i], third party payer perspective; [ii], Total health care payer perspective (in Leung et al. (2012), [ii]**+** also includes all hospital-related costs, see original article for a detailed description); [iii], societal perspective; AQOL, Australian Quality Of Life Scale; Disc. Rates, discount rates for costs and health effects, per annum; EQ-5D(-3L/5L), EuroQol 5 dimensions (3/5 levels); FTF, face-to-face; GRx, the Green Prescription; ITT, intent-to-treat analysis; LT, lifetime; n, number; (N)ERS, (national) ERS. PA, physical activity; PP, per protocol analyses; SF-36, Short Form Health Survey (36 items); T2DM, type 2 diabetes mellitus; Time hor., time horizon; UC, usual care; UC+, enhanced usual care; Y, year.

A ISO country codes.

B Mean age of total group, unless mentioned otherwise between brackets.

C Intervention versus comparator.

D As judged by the original authors, preferably based on country-specific willingness-to-pay thresholds.

E recalculation of the results (i.e. per 1,000 participants) was not possible for Cobiac et al. (2009), as it was unclear for which cohort the results were reported.

* Calculated or assumed by the authors of this review.

All studies were conducted in high-income countries^30^: New Zealand (*n* = 5) (c, f, h–j), UK (*n* = 4) (a, b, d, k–n), Australia (*n* = 2) (e, o) and Sweden (*n* = 1) (g).

Four model-based studies (j–o) and eight within-trial studies were retrieved (a–i). The majority of within-trial studies used a time horizon of 1 year (a–e, h). The time horizons for model-based studies were always lifetime. In the model-based studies, the same discount rates were applied to both costs and health outcomes: 1.5% (n), 3% (o), 3.5% (k–m) and 5% (j).

### Population

All within-trial studies had a mean baseline age between 50 and 60 years, with the exception of Leung et al. (2012) (c) with a mean baseline age of 74 years. A similar age group has been considered in the model-based studies.

To be included in this review, the study population must consist of sedentary or insufficiently active people. Two studies applied an additional criterion of having at least one (risk factor for a) medical condition (a, b, g). One study only included women (i).

### Interventions and comparators

In the majority of the studies (9/12) ERSs were compared with usual care (a, b, e–g, i–o). Three of those nine studies compared with some form of enhanced usual care. Enhanced usual care activities included providing an information leaflet highlighting the benefits of exercise, addresses of local facilities and access to ERS after the clinical trial (a, b), written information about the possibility to participate in organized PA sessions (g), or a printed pamphlet to encourage increased PA (e). The six other studies did not provide or assume any additional care for the control group.

Two of the nine studies that compared ERSs with usual care also included different delivery modes of ERSs, namely face-to-face versus telephone contact (e, o).

The three other studies only compared different delivery modes of ERSs with each other, without including usual care as an alternative. Compared delivery modes were: an ERS combined with a pedometer versus usual ERS practice (c), an ERS combined with an accelerometer versus usual ERS practice, (d), and face-to-face versus telephone contact (h).

Only Cobiac et al. (2009) (o) compared an ERS with other interventions that promote PA. The other interventions were: mass media-based campaign, pedometers and Internet-based interventions (website and/or email).

Intervention duration of the ERSs varied between 12 weeks (e) and 9 months (i), with the majority between 3 and 4 months (a–d, g–h, j, o). For two studies, the intervention duration was unclear (k–n).

### Health outcomes

Six studies expressed health gains in terms of quality-adjusted life years (QALYs) (a–e, j–m). Different instruments were used to derive utilities: EuroQol 5 Dimensions questionnaire (EQ-5D) (a–d, k–n), 36-item Short Form Health Survey (SF-36) (j) and the Australian Quality of Life scale (AQOL) (e). Cobiac et al. (2009) (o) was the only study that expressed health gains in disability-adjusted life years (DALYs) averted.

All specific or natural health outcomes were related to PA, but differed across the studies. Examples of outcomes (see also table 2) are: 30 min weekly leisure walking (c), steps per day (e), proportion of persons active (f, i), proportion being inactive (g) and days per week active (h).

### Costs

Incremental costs are shown in the evidence table (table 2). Which cost categories were included, depends from the applied perspective. In four out of eight within-trial studies (e, f, h, i) and one out of four model-based studies (j), total costs only seemed to include programme costs. The inclusion of other costs (reported as service use costs, cost offsets, cost savings) was mentioned in four out of eight within-trial studies (a, b, c, d, g) and three out of four model-based studies (k–o).

### Perspective

The majority of the studies (*n* = 9) applied a third party payer perspective, which only takes into account costs borne by the funder or national health service.

### Sensitivity analyses

Ten out of 12 studies included a one way sensitivity analysis (a–d, f, h–o), although some of them only assessed the impact of a small number of input parameters (c, d, f, i). Six out of 12 studies included a probabilistic sensitivity analysis (a–c, j–o). Input parameters related to effectiveness of ERSs often had a large impact on the cost-effectiveness results. Examples of such input parameters are: attendance less than 16 weeks (a, b); probability of becoming active (k, l); PA uptake (n); rate of decay in intervention health effect (o) or relative risk of activity gain (j).

### Critical appraisal

The critical appraisal of the individual studies is provided in table 3. More than 80% of the publications scored negative for the item ‘Research question’ (item 3). More than 80% of the publications scored positive for items ‘Competing alternatives’ (item 2), ‘study design’ (item 4), ‘cost identification’ (item 7) and ‘outcome identification’ (item 10).

**Table 3:** Quality assessment of all included publications, sorted from highest percentage score (left) to lowest percentage score (right)

		Cobiac (2009)	Dalziel (2005)	Leung (2012)	Anokye (2011)	Edwards (2013)	Elley (2011)	Murphy (2012)	Foley (2011)	Pavey (2011)	Campbell (2015)	Romé (2009)	Elley (2004)	Trueman (2012)	Hawkins (2019)	Ewald (2018)
1	Study population	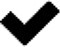	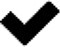	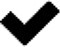	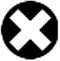	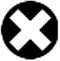	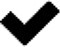	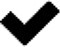	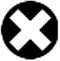	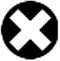	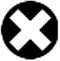	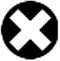	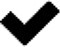	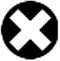	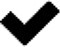	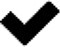
2	Competing alternatives	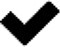	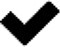	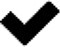	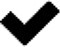	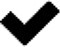	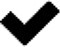	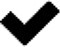	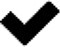	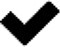	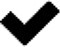	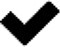	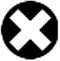	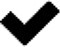	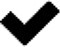	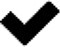
3	Research question	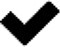	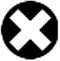	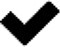	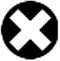	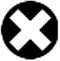	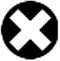	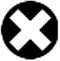	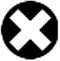	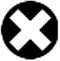	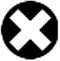	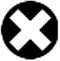	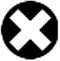	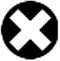	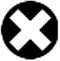	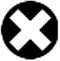
4	Study design	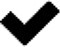	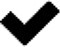	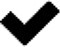	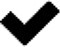	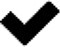	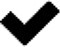	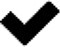	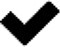	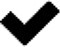	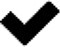	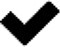	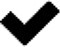	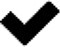	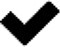	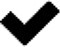
5	Time horizon	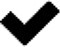	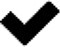	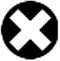	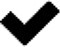	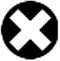	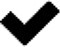	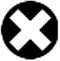	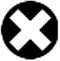	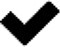	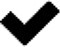	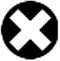	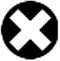	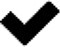	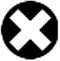	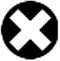
6	Perspective	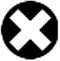	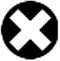	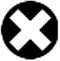	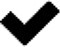	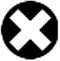	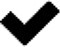	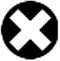	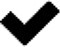	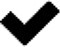	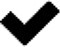	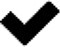	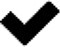	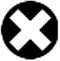	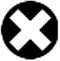	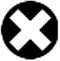
7	Cost identification	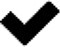	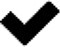	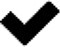	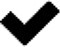	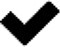	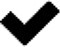	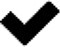	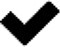	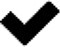	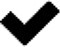	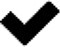	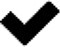	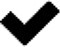	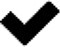	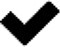
8	Cost measurement	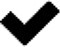	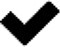	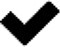	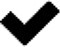	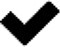	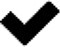	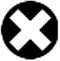	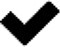	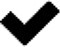	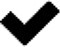	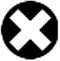	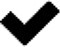	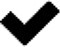	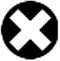	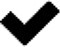
9	Cost valuation	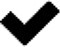	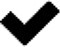	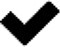	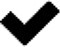	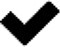	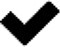	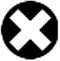	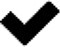	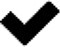	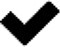	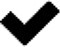	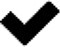	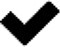	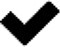	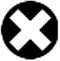
10	Outcome identification	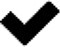	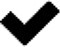	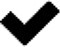	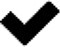	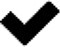	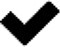	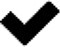	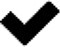	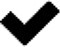	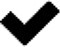	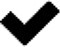	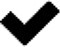	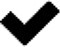	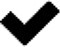	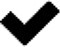
11	Outcome measurement	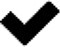	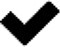	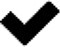	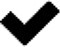	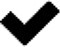	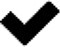	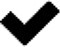	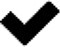	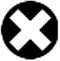	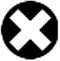	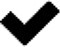	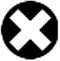	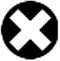	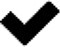	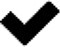
12	Outcome valuation	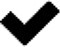	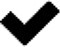	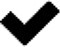	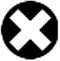	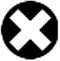	n.a.	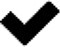	n.a.	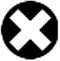	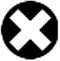	n.a.	n.a.	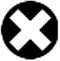	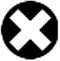	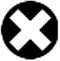
13	Incremental analysis	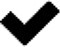	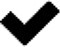	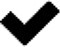	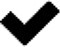	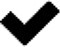	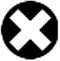	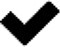	n.a.	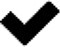	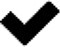	n.a.	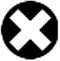	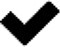	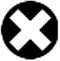	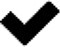
14	Discounting	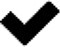	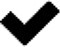	n.a.	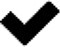	n.a.	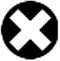	n.a.	n.a.	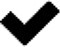	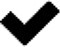	n.a.	n.a.	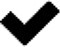	n.a.	n.a.
15	Sensitivity analysis	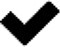	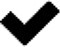	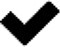	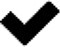	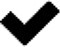	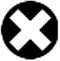	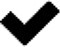	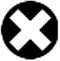	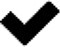	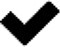	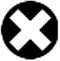	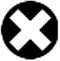	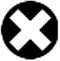	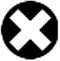	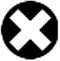
16	Conclusions	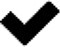	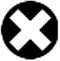	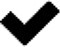	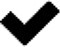	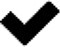	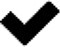	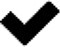	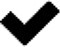	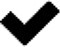	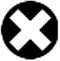	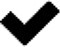	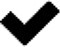	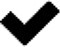	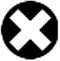	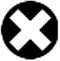
17	Generalizability	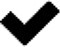	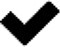	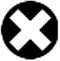	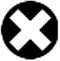	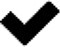	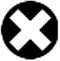	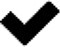	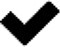	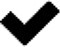	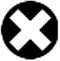	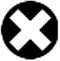	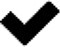	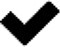	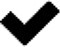	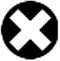
18	No conflict of interest	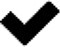	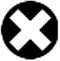	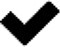	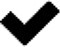	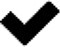	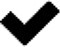	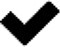	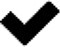	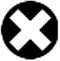	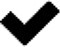	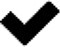	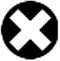	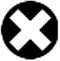	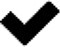	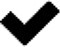
19	Ethics	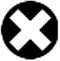	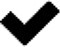	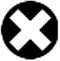	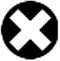	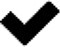	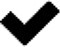	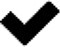	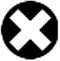	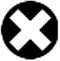	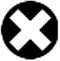	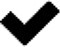	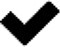	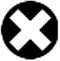	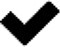	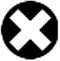
		17/19	15/19	14/18	14/19	13/18	13/18	13/18	11/16	13/19	12/19	10/16	10/17	11/19	10/18	9/18
		89%	79%	78%	74%	72%	72%	72%	69%	68%	63%	63%	59%	58%	56%	50%

*Notes*: 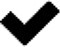 Sufficient attention is given to this aspect. 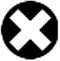 Insufficient attention is given to this aspect. n.a., not applicable.

### Synthesis of results

No differences in results were found for studies that compared ERSs to usual care and studies that compared with enhanced usual care. Hence, no further distinction between these two was made in the further syntheses of results. ERSs were considered to be cost-effective compared with (enhanced) usual care in a majority of the analyses. Ten analyses aimed to express the results by means of an ICER, out of which seven analyses showed that the ICER was below a given threshold, thus considered ERSs cost-effective compared with (enhanced) usual care (a, b, e, f, i–m, o). However, relatively modest health gains were reported, both in terms of natural health outcomes as well as QALY gains. For example, when comparing with (enhanced) usual care, the highest QALY gains reported—over a lifetime time horizon—were 79 QALYs per 1,000 persons (j). However, similar to health gains, incremental costs were also relatively small.

Four studies compared different types of ERSs to each other. Based on two studies, an ERS combined with a pedometer or accelerometer was cost-effective compared with a usual ERS practice (c, d). Three other studies compared phone-based with face-to-face ERSs (e, h, o). Two out of three studies, Ewald et al. (2008) and Foley et al (2011), found that phone-based and face-to-face ERSs were equally effective, with similar costs (e, h). The third study, Cobiac et al. (2009), found that a phone-based ERS compared with usual care was cost-effective, while a face-to-face ERS compared with usual care was not cost-effective (o). The latter study also included other interventions that promote PA. Pedometers, mass media-based campaigns and an Internet-based intervention appeared to provide more cost-effective results than the two types of ERSs that were included in this study (o).

## Discussion

The primary aim of this systematic review was to provide an overview of the existing literature on cost-effectiveness of ERSs for insufficiently active people. ERSs were found to be cost-effective compared with (enhanced) usual care in a majority of the analyses, but with relatively modest health gains and costs. However, small health gains are certainly not atypical for public health interventions.^31^ Nevertheless, this also implies that the ICERs are very sensitive to small changes in health gains or incremental costs. Hence, conclusions drawn from the ICERs could easily change due to a small increase or decrease in input parameter values. This was confirmed by the sensitivity analyses in several included studies, which often showed a large impact of input parameters related to effectiveness of ERS. Additionally, as mentioned in the ‘Introduction’, evidence about the effectiveness of ERSs appears to be inconsistent.^12^ Low attendance rates in ERSs have been reported, which could at least partially explain the inconsistencies in the literature regarding effectiveness.^12^^,^^32^ Another possible explanation are the various intervention durations. Overall, follow-up periods of clinical trials might be too short to assess if ERSs can lead to a sustainable behavioural change towards an active lifestyle. Hence, given the uncertainty about data inputs such as (long-term) effectiveness of and/or attendance to ERSs, and given their impact on results in health-economic evaluations, conclusions regarding cost-effectiveness should be interpreted with caution.

Few studies compared the cost-effectiveness of different delivery modes of ERSs. Two studies found that ERS combined with a pedometer or accelerometer was more effective and also cost-effective, compared with usual ERS practice. This could be explained by (additional) behavioural change techniques.^33^^,^^34^ First, the use of pedometers or accelerometers can allow users to set other goals (next to time-based goals), such as a number of steps per day. Second, providing feedback or self-monitoring is another behavioural change technique that is often applied to increase PA, for instance in mobile applications.^35^ On the other hand, Hawkins et al. (2019) mentioned that high attrition is common when technological devices are used, with only 10% of the participants still engaging with the device by the end of the study. If device usage would decrease even faster in a real-life setting, ERSs combined with a device might not be as cost-effective as the studies in this review suggest.

Three studies assessed ERSs via phone support versus ERSs via face-to-face support, of which two of those found that these delivery modes were equally effective, with similar costs. Attendance and/or adherence is again one of the key aspects discussed by the original authors. For example, Foley et al. (2011) pointed out that providing a choice to the participants about the delivery mode might positively affect attendance. Edwards et al. (2016) (b) stated that an ERS is likely to be cost-saving in participants who completed the programme. This suggests that efforts should be made to affect people’s PA behaviour and maintenance of their behaviour. In that case, these additional efforts to increase attendance might ask for more time and effort from the coaches and/or the organization, which could induce additional costs. Depending on the chosen perspective, this can affect the cost-effectiveness of the intervention. The use of mobile applications might provide opportunities to increase attendance and adherence at a reasonable cost. Lower costs have been reported with the use of m-health.^36^ Looking back at our results, it is important to note that both phone-based and face-to-face ERSs can be seen as two delivery methods that still allow for an individualized approach. A blended approach, in which an individualized trajectory is first discussed with a health-care provider (either face-to-face or via telephone) and monitoring and follow-up is afterwards achieved via a mobile application, could possibly combine the best of both worlds. The impact of additional efforts and/or tools to improve attendance and adherence on the programme costs and effects should be subject to further research.

The majority of the studies applied a third party payer perspective. This is because some national guidelines recommend this perspective,^29^ but also because broader perspectives require more data collection on costs and/or more cost calculations, which is often challenging or even unfeasible. However, the perspective has a large impact on cost-effectiveness results, and it can be expected that the adoption of a broader perspective will lead to a more cost-effective result.^16^

Based on our quality assessment, a clear research question was almost always lacking in the original publications. Although it might feel as obvious or unnecessary repetition, the inclusion of a clear research question in a standardized form is of added value for readers. According to the assessment instruction of the CHEC-list, the research question should include the alternatives being compared and the population for which the comparison is made.^27^ Aside from the research question, studies showed a good methodological quality. This could be explained by a close cooperation between health economic researchers and PA researchers, as shown by the affiliations of the authors. Interdisciplinary collaborations pay off and should be further encouraged, for example by funding organizations.

### Limitations

In previous systematic reviews or discussions of original studies included in the current review, other health economic evaluations of ERSs were mentioned.^37^^,^^38^ However, these are not included in our review, as the assessed interventions did not meet our definition of ERS. More specifically, the exclusion criterion for interventions that were not tailored or individualized appeared to be crucial. As this is an important characteristic of an ERS, and eligibility criteria were predefined and should not be altered throughout the review process, it was preferred not to alter this criterion.

## Conclusions

ERSs were found to be cost-effective compared with (enhanced) usual care in a majority of the studies. However, health gains and incremental costs were small and ICERs were very sensitive to small changes in the input parameters, implying that cost-effectiveness results of ERSs compared with usual care are not robust. Studies that compared different delivery methods of ERSs showed the potential impact of behavioural change techniques such as goal-setting, self-monitoring and feedback. Additionally, providing a choice to the participants about the delivery mode might positively affect attendance rates and as such effectiveness and cost-effectiveness of ERS. For further research, there is need for clarity on effectiveness of and attendance to ERS. More certainty about these key input parameters will strengthen health-economic evidence, and thus will allow to provide a clearer message to health care policy makers.

## Supplementary data

Supplementary data are available at *EURPUB* online.

## Funding

The project was funded by ‘Vlaams Agentschap voor Zorg en Gezondheid’ (VAZG, The Flemish Agency for Care and Health, AZG/PREV/GE/2016-01). The funder was involved in the selection of the topic but had no role in study design, data collection, data analysis, data interpretation or reporting.

*Conflicts of interest*: None declared.

Key points

- In comparison with usual care, ERSs were found to be cost-effective in a majority of the included analyses.
- The results were not robust. In other words, small changes in health gains or incremental costs may have a large impact on the results and conclusions.
- Overall, the methodological quality of the included studies was good.
- There is a need for further research on effectiveness of and attendance to ERSs.

## Supplementary Material

ckab189_Supplementary_Data
