# Supplementary material for: Cost-effectiveness of exercise referral schemes: a systematic review of health economic studies
Source: Eur J Public Health. 2021 Dec 4;32(1):87–94. doi: 10.1093/eurpub/ckab189 (PMC9090165; doi:10.1093/eurpub/ckab189)
Supplement: ckab189_Supplementary_Data [file ckab189_supplementary_data.zip › ejph-2020-05-om-0590-File006.docx]

Search strategies for all the databases

All search strategies were performed on October 16^th^, 2020.

# MEDLINE (via PubMed)

|  | **Search terms** | **No. of hits** |
| --- | --- | --- |
| I | (physical activit*[tiab] OR "Exercise Therapy"[Mesh] OR "Exercise"[Mesh] OR exercise*[tiab]) |  |
|  | ("Prescriptions"[Mesh] OR prescrib*[tiab] OR prescription[tiab] OR "Referral and Consultation"[Mesh] OR referr*[tiab]) |  |
|  | #1 AND #2 |  |
|  | “green prescription”[tiab] OR "exercise is medicine"[tiab] |  |
|  | #3 OR #4 |  |
| O | "Cost-Benefit Analysis"[Mesh] |  |
|  | economic evaluation*[tiab] OR economic analy*[tiab] |  |
|  | health economic[tiab] OR health-economic[tiab] OR healtheconomic[tiab] |  |
|  | cost analy*[tiab] OR cost-analy*[tiab] OR costanaly*[tiab] |  |
|  | cost effective[tiab] OR cost-effective[tiab] OR costeffective[tiab] OR cost effectiveness[tiab] OR cost-effectiveness[tiab] OR costeffectiveness[tiab] |  |
|  | cost efficien*[tiab] OR cost-efficien*[tiab] OR costefficien*[tiab] |  |
|  | cost benefit*[tiab] OR cost-benefit*[tiab] OR costbenefit*[tiab] |  |
|  | cost utilit*[tiab] OR cost-utilit*[tiab] OR costutilit*[tiab] |  |
|  | cost minimization[tiab] OR cost-minimization[tiab] OR costminimization[tiab] |  |
|  | value for money[tiab] |  |
|  | "Quality-Adjusted Life Years"[Mesh] |  |
|  | qaly*[tiab] OR ((quality adjusted[tiab] OR quality-adjusted[tiab] OR qualityadjusted[tiab]) AND (year[tiab] OR years[tiab])) |  |
|  | #6 OR #7 OR #8 OR #9 OR #10 OR #11 OR #12 OR #13 OR #14 OR #15 OR #16 OR #17 |  |
| I+O | #5 AND #18 | 528 |

I: Intervention; O: Outcome; [Mesh]: Mesh term (PubMed subject headings); [tiab]: free terms searched in title, abstract and keywords; *: wildcard.

# EMBASE (via embase.com)

|  | Search terms | No. of hits |
| --- | --- | --- |
| I | ('physical activity, capacity and performance'/exp OR 'physical activit*':ti,ab,kw OR exercise:ti,ab,kw) |  |
|  | ('prescription'/exp OR prescription:ti,ab,kw OR prescrib*:ti,ab,kw OR 'patient referral'/exp OR referr*:ti,ab,kw) |  |
|  | #1 AND #2 |  |
|  | green prescription':ti,ab,kw OR 'exercise is medicine':ti,ab,kw |  |
|  | #3 OR #4 |  |
| O | cost benefit analysis'/exp OR 'cost effectiveness analysis'/exp OR 'cost minimization analysis'/exp OR 'cost utility analysis'/exp |  |
|  | economic evaluation*':ti,ab,kw OR 'economic analy*':ti,ab,kw |  |
|  | health economic':ti,ab,kw OR 'health-economic':ti,ab,kw OR healtheconomic:ti,ab,kw |  |
|  | cost analy*':ti,ab,kw OR 'cost-analy*':ti,ab,kw OR costanaly*:ti,ab,kw |  |
|  | cost effective':ti,ab,kw OR 'cost-effective':ti,ab,kw OR costeffective:ti,ab,kw OR 'cost effectiveness':ti,ab,kw OR 'cost-effectiveness':ti,ab,kw OR costeffectiveness:ti,ab,kw |  |
|  | cost efficien*':ti,ab,kw OR 'cost-efficien*':ti,ab,kw OR costefficien*:ti,ab,kw |  |
|  | cost benefit':ti,ab,kw OR 'cost-benefit':ti,ab,kw OR costbenefit:ti,ab,kw |  |
|  | cost utilit*':ti,ab,kw OR 'cost-utilit*':ti,ab,kw OR costutilit*:ti,ab,kw |  |
|  | cost minimization':ti,ab,kw OR 'cost-minimization':ti,ab,kw OR costminimization:ti,ab,kw |  |
|  | value for money':ti,ab,kw |  |
|  | quality adjusted life year'/exp |  |
|  | qaly*':ti,ab,kw OR (('quality adjusted':ti,ab,kw OR 'quality-adjusted':ti,ab,kw OR qualityadjusted:ti,ab,kw) AND (year:ti,ab,kw OR years:ti,ab,kw)) |  |
|  | #6 OR #7 OR #8 OR #9 OR #10 OR #11 OR #12 OR #13 OR #14 OR #15 OR #16 OR #17 |  |
| I+O | #5 AND #18 |  |
|  | #19 NOT 'conference abstract'/it | 927 |

I: Intervention; O: Outcome; /exp: Emtree term (Embase subject headings); ti,ab,kw: free terms searched in title, abstract and keywords; *: wildcard.

# EconLit search

|  | **Search terms** | **No. of hits** |
| --- | --- | --- |
| I | noft("physical activit*" OR "exercise") |  |
|  | noft("prescription" OR "prescrib*" OR "referr*") |  |
|  | #1 AND #2 |  |
|  | noft("green prescription" OR "exercise is medicine") |  |
|  | #3 OR #4 | 89 |

I: Intervention; noft: free terms searched anywhere except full text;*: wildcard.

# PsycINFO search

|  | Search terms | No. of hits |
| --- | --- | --- |
| I | noft("physical activities" OR "physical activity" OR "exercise") |  |
|  | noft(prescrib* OR "referr*") |  |
|  | S1 AND S2 |  |
|  | noft("green prescription" OR "exercise is medicine") |  |
|  | S3 OR S4 |  |
| O | noft("economic evaluation*" OR "economic analy*") |  |
|  | noft("health economic" OR "health-economic" OR "healtheconomic") |  |
|  | noft("cost analy*" OR "cost-analy*" OR "costanaly*") |  |
|  | noft("cost effective" OR "cost-effective" OR "costeffective" OR "cost effectiveness" OR "cost-effectiveness" OR "costeffectiveness") |  |
|  | noft("cost efficien*" OR "cost-efficien*" OR "costefficien*") |  |
|  | noft("cost benefit" OR "cost-benefit" OR "costbenefit") |  |
|  | noft("cost utilit*" OR "cost-utilit*" OR "costutilit*") |  |
|  | noft("cost minimization" OR "cost-minimization" OR "costminimization") |  |
|  | noft("value for money") |  |
|  | noft("qaly*" OR (("quality adjusted" OR "quality-adjusted" OR "qualityadjusted") AND ("year" OR "years"))) |  |
|  | S6 OR S7 OR S8 OR S9 OR S10 OR S11 OR S12 OR S13 OR S14 OR S15 |  |
|  | S5 AND S16 | 39 |

I: Intervention; noft: free terms searched anywhere except full text;*: wildcard.

# Web of Science search

|  | Search terms | No. of hits |
| --- | --- | --- |
| I | TS=("physical activit*" OR "exercise") |  |
|  | TS=("prescription" OR "prescrib*" OR "referr*") |  |
|  | #1 AND #2 |  |
|  | TS=("green prescription" OR "exercise is medicine") |  |
|  | #3 OR #4 |  |
| O | TS=("economic evaluation*" OR "economic analy*") |  |
|  | TS=("health economic" OR "health-economic" OR "healtheconomic") |  |
|  | TS=("cost analy*" OR "cost-analy*" OR "costanaly*") |  |
|  | TS=("cost effective" OR "cost-effective" OR "costeffective" OR "cost effectiveness" OR "cost-effectiveness" OR "costeffectiveness") |  |
|  | TS=("cost efficien*" OR "cost-efficien*" OR "costefficien*") |  |
|  | TS=("cost benefit" OR "cost-benefit" OR "costbenefit") |  |
|  | TS=("cost utilit*" OR "cost-utilit*" OR "costutilit*") |  |
|  | TS=("cost minimization" OR "cost-minimization" OR "costminimization") |  |
|  | TS=("value for money") |  |
|  | TS=("qaly*" OR (("quality adjusted" OR "quality-adjusted" OR "qualityadjusted") AND ("year" OR "years"))) |  |
|  | #6 OR #7 OR #8 OR #9 OR #10 OR #11 OR #12 OR #13 OR #14 OR #15 |  |
| I+O | #5 AND #16 | 499 |

I: Intervention; O: Outcome; TS: topic terms; *: wildcard.
