# Supplementary material for: Cost-effectiveness of exercise referral schemes: a systematic review of health economic studies
Source: Eur J Public Health. 2021 Dec 4;32(1):87–94. doi: 10.1093/eurpub/ckab189 (PMC9090165; doi:10.1093/eurpub/ckab189)
Supplement: ckab189_Supplementary_Data [file ckab189_supplementary_data.zip › ejph-2020-05-om-0590-File008.docx]

1. Murphy SM, Edwards RT, Williams N, Raisanen L, Moore G, Linck P, et al. An evaluation of the effectiveness and cost effectiveness of the National Exercise Referral Scheme in Wales, UK: a randomised controlled trial of a public health policy initiative. Journal of epidemiology and community health. 2012;66(8):745-53.
2. Edwards RT, Linck P, Hounsome N, Raisanen L, Williams N, Moore L, et al. Cost-effectiveness of a national exercise referral programme for primary care patients in Wales: results of a randomised controlled trial. BMC Public Health. 2013;13:1021.
3. Leung W, Ashton T, Kolt GS, Schofield GM, Garrett N, Kerse N, et al. Cost-effectiveness of pedometer-based versus time-based Green Prescriptions: the Healthy Steps Study. Australian journal of primary health. 2012;18(3):204-11.
4. Hawkins J, Charles JM, Edwards M, Hallingberg B, McConnon L, Edwards RT, et al. Acceptability and Feasibility of Implementing Accelorometry-Based Activity Monitors and a Linked Web Portal in an Exercise Referral Scheme: Feasibility Randomized Controlled Trial. Journal of medical Internet research. 2019;21(3):e12374.
5. Ewald B, Stacey F, Johnson N, Plotnikoff RC, Holliday E, Brown W, et al. Physical activity coaching by Australian Exercise Physiologists is cost effective for patients referred from general practice. Australian and New Zealand journal of public health. 2018;42(1):12-5.
6. Elley R, Kerse N, Arroll B, Swinburn B, Ashton T, Robinson E. Cost-effectiveness of physical activity counselling in general practice. The New Zealand medical journal. 2004;117(1207):U1216.
7. Rome A, Persson U, Ekdahl C, Gard G. Physical activity on prescription (PAP): costs and consequences of a randomized, controlled trial in primary healthcare. Scandinavian journal of primary health care. 2009;27(4):216-22.
8. Foley L, Maddison R, Jones Z, Brown P, Davys A. Comparison of two modes of delivery of an exercise prescription scheme. The New Zealand medical journal. 2011;124(1338):44-54.
9. Elley CR, Garrett S, Rose SB, O'Dea D, Lawton BA, Moyes SA, et al. Cost-effectiveness of exercise on prescription with telephone support among women in general practice over 2 years. British journal of sports medicine. 2011;45(15):1223-9.
10. Dalziel K, Segal L, Elley CR. Cost utility analysis of physical activity counselling in general practice. Australian and New Zealand journal of public health. 2006;30(1):57-63.
11. Anokye NK, Trueman P, Green C, Pavey TG, Hillsdon M, Taylor RS. The cost-effectiveness of exercise referral schemes. BMC Public Health. 2011;11:954.
12. Pavey TG, Anokye N, Taylor AH, Trueman P, Moxham T, Fox KR, et al. The clinical effectiveness and cost-effectiveness of exercise referral schemes: a systematic review and economic evaluation. Health technology assessment (Winchester, England). 2011;15(44):i-xii, 1-254.
13. Trueman P, Anokye NK. Applying economic evaluation to public health interventions: the case of interventions to promote physical activity. Journal of public health (Oxford, England). 2013;35(1):32-9.
14. Campbell F, Holmes M, Everson-Hock E, Davis S, Buckley Woods H, Anokye N, et al. A systematic review and economic evaluation of exercise referral schemes in primary care: a short report. Health technology assessment (Winchester, England). 2015;19(60):1-110.
15. Cobiac LJ, Vos T, Barendregt JJ. Cost-effectiveness of interventions to promote physical activity: a modelling study. PLoS Med. 2009;6(7):e1000110.
